# When all else fails: the colo-duodenal anastomosis

**DOI:** 10.1093/jscr/rjae137

**Published:** 2024-03-11

**Authors:** Suzannah L Dewhurst, Mina Sarofim, Ruwanthi Wijayawardana, David L Morris

**Affiliations:** Hepatobiliary and Surgical Oncology Unit, Department of Surgery, St George Hospital, Sydney 2217, Australia; Hepatobiliary and Surgical Oncology Unit, Department of Surgery, St George Hospital, Sydney 2217, Australia; Hepatobiliary and Surgical Oncology Unit, Department of Surgery, St George Hospital, Sydney 2217, Australia; Hepatobiliary and Surgical Oncology Unit, Department of Surgery, St George Hospital, Sydney 2217, Australia

**Keywords:** total enterectomy, duodenocolonic anastomosis, parental nutrition

## Abstract

Total enterectomy is an exceedingly rare procedure performed out of necessity due to massive intestinal infarction, trauma, or peritoneal malignancy. This case describes a 47-year-old patient who has successfully undergone the procedure to manage mesenteric ischaemia. Bowel continuity was achieved with a duodenocolonic anastomosis, and the patient has been transitioned to life-long total parental nutrition. This case highlights that carefully selected patients can achieve long-term survival with good quality of life rather than palliation.

## Introduction

Total enterectomy is a rare procedure associated with high-risk of mortality and morbidity. It is performed out of necessity to manage life-threatening pathologies including mesenteric ischaemia, trauma, and malignancy. This case describes the successful management of a patient who underwent total enterectomy to treat mesenteric ischaemia.

## Case report

A 47-year-old female presented to a tertiary hospital with a 5-day history of generalized abdominal pain. She reported anorexia, diarrhoea, and fevers. She was a non-smoker with no cardiorespiratory comorbidities.

**Figure 1:**
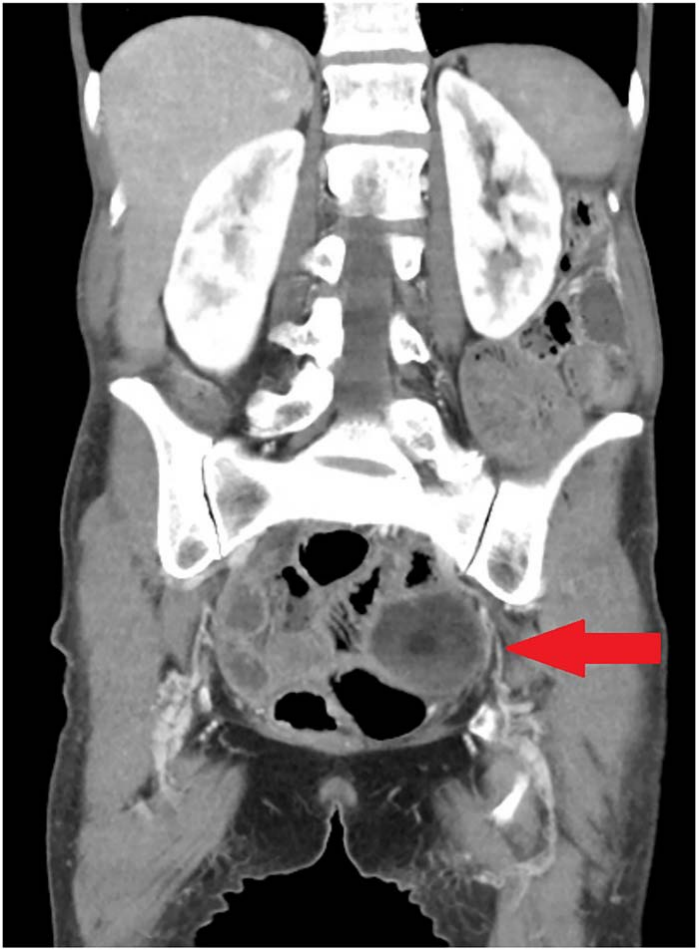
Coronal view of initial CT scan demonstrating left adnexal dermoid cyst (arrow).

On examination she had a diffusely tender abdomen, with maximal tenderness in her left lower quadrant. Her neutrophils were 16x10^9^/L and C reactive protein 98 mg/L. Computed tomography (CT) imaging of the abdomen and pelvis demonstrated patchy mucosal enhancement of the right colon in addition to a complex left-sided dermoid cyst (Fig. 1), which was confirmed on pelvic ultrasound.

**Figure 2:**
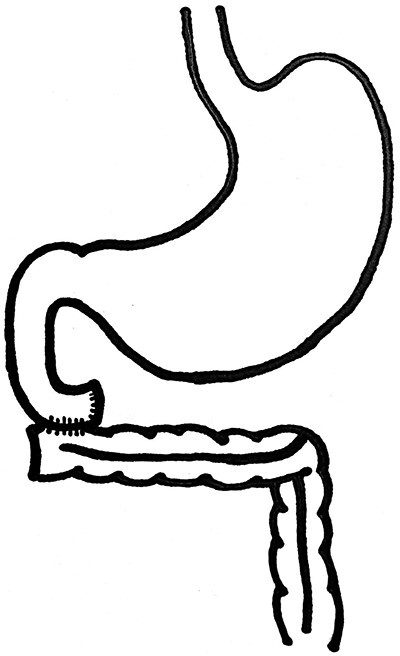
Diagrammatic representation of the handsewn two-layer duodenocolonic anastomosis.

The patient underwent a laparoscopic left oophorectomy and salpingectomy by the gynaecological surgeons for the complex ovarian lesion. Day 1 post-operatively the patient’s pain was not improved, and she returned to the operating theatre for laparotomy which revealed ischaemic small bowel and caecum, and pulseless coeliac and superior mesenteric arteries. Intra-operatively the general and vascular surgeons deemed no role for embolectomy, and a total enterectomy was performed from duodenojejunal flexure to the mid-transverse colon, and a laparostomy with negative pressure applied. Following a family meeting to assess social support and wishes, the patient underwent re-look laparotomy and washout 24-h later. Gastrointestinal continuity was established by a handsewn two-layer anastomosis between mid-transverse colon and the duodenum (Fig. 2).

Post-operatively the patient made a steady recovery but required a prolonged admission to manage short bowel syndrome (SBS) and transitioning to life-long total parental nutrition (TPN). At 2 year follow-up she remains fully independent at home with stable body mass index, but has required hospitalization for intravenous antibiotic treatment of central line sepsis.

## Discussion

Enterectomy, or total resection of the jejunum and ileum, is an exceedingly rare and radical procedure performed out of necessity due to massive intestinal infarction, trauma, or peritoneal malignancy. Fatal short-term complications in addition to long-term malabsorption due to SBS and intestinal failure make total enterectomy a high-risk operation. Haemodynamic instability, age, comorbidities, or a patient and family decision to discontinue care, means patients with global small bowel ischaemia are commonly palliated. Rarely, a haemodynamically stable patient will undergo total enterectomy.

Recent reviews and institutional studies demonstrate ~45 patients worldwide who have successfully undergone total enterectomy [1–3]. Surgical technique for reconstruction is a challenge and varies greatly between cases, likely due to extremely low case numbers and the unique circumstances of each surgical emergency. Reported cases include gastrocolonic or duodenocolonic anastomoses, with or without a gastrostomy tube to manage gastric secretions. Alternatively, duodenostomy has been described when there is sufficient duodenal length [3], or gastroduodenostomy with external drainage when there is not [4].

When possible, reconstruction to establish bowel continuity is the preferred surgical method for two main reasons. Firstly, there is a high risk of fistulization of duodenal stumps due to biliopancreatic secretions [2]. Secondly, compared with ostomy patients, continuity with the colon has been demonstrated to reduce fluid and electrolyte requirements by ~25%, as well as parenteral energy requirements [3]. It has been shown that if 75 cm of small bowel is in continuity with the colon, patients are able to substantially wean their TPN requirement [5].

TPN is the mainstay of treatment for patients with intestinal failure due to SBS, impaired intestinal motility or mucosal function, which includes patients with Crohn’s disease, radiation enteritis, systemic sclerosis, or active malignancy [6]. Patients with SBS have demonstrated favourable TPN morbidity and mortality outcomes compared with those with other underlying pathologies. Poorer prognosis for patients with obstruction or gastrointestinal dysfunction is thought to be related to complications of the underlying disease [7].

Life-long TPN is associated with high morbidity primarily due to hepatobiliary complications, line-associated sepsis and thrombosis, or bone and electrolyte abnormalities [1]. Hepatobiliary complications are multifactorial; fatty acid derivatives lead to oxidative stress and systemic inflammation, while bowel rest allows for bacterial overgrowth—both of which contribute to cholestasis and steatosis [8]. Fat emulsions in TPN have been identified as a risk factor for cholestasis, hence more recent TPN formulations use a lipid dose of <1 mg/kg with good effect [8]. Cholestasis and steatosis if not monitored may lead to fibrosis and subsequently cirrhosis. Incidence of fulminant liver failure is reportedly low, however it carries an extremely high mortality rate [9].

For those on long-term TPN, the annual incidence of central line infection or thrombosis is ~0.25% and 0.07%, respectively [6, 10, 11]. The likelihood of line sepsis correlates with duration of treatment, and although it has a low mortality rate, line sepsis accounts for a considerable number of hospital admissions for TPN patients [6]. The incidence of metabolic bone disease is 0.06% annually, and electrolyte abnormalities 0.5% per year [1].

Quality of life (QOL) on parenteral nutrition is a major consideration in surgical decision making for surgeons, patients, and families. Reduced QOL for patients correlates to fatigue, reduced oral intake, and the social burden of the time intensive treatment [12]. Since TPN relies heavily on the personal responsibility of the patient to administer and maintain sterility, it is of vital importance to recognize and address the social implications of the treatment [13].

This is an extremely rare case which reports the successful management of a patient following enterectomy due to mesenteric ischaemia. It highlights that carefully selected patients can achieve long-term survival with good QOL rather than palliation, if complications such as line-sepsis and malnutrition are well managed.

## References

[1] Huerta S , Kukreja S, Carter K, Butler D. No gut syndrome: near total enterectomy. J Gastrointest Surg 2015;19:973–80. 10.1007/s11605-015-2787-2. 25791906

[2] Cruz RJ , Butera L, Poloyac K, McGurgan J, Stein W, Binion D, et al. Surgical and medical approach to patients requiring total small bowel resection: managing the no gut syndrome. Surgery 2017;162:871–9. 28755968 10.1016/j.surg.2017.05.012

[3] Cruz R , Mcgurgan J, Stein W, Gunabushanam V, Khanna A, Butera L, et al. Management of duodenal stump after total enterectomy. Is there any nutritional benefit of gastrointestinal reconstruction in patients with ‘no gut syndrome’? Transplantation 2023;107:20. 10.1097/01.tp.0000945596.88633.49.

[4] Hofker TO, Kaijser MA, Nieuwenhuijs VB, Lange JFM, Hofker HS. Distal duodenogastrostomy or proximal jejunogastrostomy in the management of ultra-short bowel. J Gastrointest Surg 2018;22:538–43. 10.1007/s11605-017-3654-0. 29273999 PMC5838119

[5] Lauro A , Cirocchi R, Cautero N, Dazzi A, Pironi D, Di Matteo F, et al. Reconnection surgery in adult post-operative short bowel syndrome <100cm: is colonic continuity sufficient to achieve enteral autonomy without autologous gastrointestinal reconstruction? Report from a single centre and systematic review of literature. J Ital Surg Assoc 2017;38:163–75. 10.11138/gchir/2017.38.4.163 PMC5725160 29182898

[6] Lloyd DAJ , Vega R, Bassett P, Forbes A, Gabe SM. Survival and dependence on home parenteral nutrition: experience over a 25-year period in a UK referral centre. Aliment Pharmacol Ther 2006;24:1231–40. 10.1111/j.1365-2036.2006.03106.x. 17014582

[7] Mejías Trueba M , Rodríguez Ramallo H, Seisdedos Elcuaz R, Pérez Blanco JL, García Luna PP, Serrano Aguayo P, et al. Our eight-year experience in home parenteral nutrition for adult patients. Nutr Hosp 2020;37:654–9. 10.20960/nh.03008 32686441

[8] Mitra A , Ahn J. Liver Disease in Patients on Total Parenteral Nutrition. Clinics in Liver Disease 2017;21:687–95. 10.1016/j.cld.2017.06.008. 28987256

[9] Chan S , McCowen KC, Bistrian BR, Thibault A, Keane-Ellison M, Forse RA, et al. Incidence, prognosis, and etiology of end-stage liver disease in patients receiving home total parenteral nutrition. Surgery 1999;126:28–34. 10.1067/msy.1999.98925. 10418589

[10] Vantini I , Benini L, Bonfante F, Talamini G, Sembenini C, Chiarioni G, et al. Survival rate and prognostic factors in patients with intestinal failure. Dig Liver Dis 2004;36:46–55. 10.1016/j.dld.2003.09.015. 14971815

[11] Pironi L , Paganelli F, Labate AMM, Merli C, Guidetti C, Spinucci G, et al. Safety and efficacy of home parenteral nutrition for chronic intestinal failure: a 16-year experience at a single centre. Dig Liver Dis 2003;35:314–24. 10.1016/S1590-8658(03)00074-4. 12846403

[12] Huisman-de Waal G , Schoonhoven L, Jansen J, Wanten G, van Achterberg T. The impact of home parenteral nutrition on daily life—a review. Clin Nutr 2007;26:275–88. 10.1016/j.clnu.2006.10.002. 17161888

[13] Schönenberger KA , Reber E, Huwiler VV, Dürig C, Muri R, Leuenberger M, et al. Quality of life in the Management of Home Parenteral Nutrition. Ann Nutr Metab 2023;79:326–33. 10.1159/000530082. 36934718 PMC10614234

